# Knockdown of PAICS inhibits malignant proliferation of human breast cancer cell lines

**DOI:** 10.1186/s40659-018-0172-9

**Published:** 2018-08-10

**Authors:** Minjun Meng, Yanling Chen, Jianbo Jia, Lianghui Li, Sumei Yang

**Affiliations:** 1 Department of Breast Surgery, The Affiliated Zhongshan Hospital of Xiamen University, No. 201, Hubin South Road, Xiamen, 361000 Fujian China; 2 Xiamen Siming District Kaiyuan Street Community Health Service Center, Xiamen, 361000 Fujian China

**Keywords:** Breast cancer, PAICS, Proliferation, Cell cycle, Cell apoptosis

## Abstract

**Background:**

Phosphoribosylaminoimidazole carboxylase, phosphoribosylaminoimidazole succinocarboxamide synthetase (PAICS), an enzyme required for de novo purine biosynthesis, is associated with and involved in tumorigenesis. This study aimed to evaluate the role of PAICS in human breast cancer, which remains the most frequently diagnosed cancer and the leading cause of cancer-related death among women in less developed countries.

**Results:**

Lentivirus-based short hairpin RNA targeting PAICS specifically depleted its endogenous expression in ZR-75-30 and MDA-MB-231 breast cancer cells. Depletion of PAICS led to a significant decrease in cell viability and proliferation. To ascertain the mechanisms through which PAICS modulates cell proliferation, flow cytometry was performed, and it was confirmed that G1-S transition was blocked in ZR-75-30 cells through PAICS knockdown. This might have occurred partly through the suppression of Cyclin E and the upregulation of Cyclin D1, P21, and CDK4. Moreover, PAICS knockdown obviously promoted cell apoptosis in ZR-75-30 cells through the activation of PARP and caspase 3 and downregulation of Bcl-2 and Bcl-xl expression in ZR-75-30 cells.

**Conclusions:**

These findings demonstrate that PAICS plays an essential role in breast cancer proliferation in vitro, which provides a new opportunity for discovering and identifying novel effective treatment strategies.

## Background

De novo purine biosynthesis pathway is ubiquitous across all living species and catalyzes the conversion of phosphoribosyl pyrophosphate to inosine monophosphate. It has become clear that rapidly dividing cancer cells rely heavily on the de novo purine pathway for the synthesis of adenine and guanine, whereas normal cells recover purines via the salvage pathway [1]. Further research has indicated that inactivation of the de novo pathway (e.g. by the anti-folate drug methotrexate) inhibits malignant cancer cell proliferation both in vitro and in vivo [2]. Therefore, enzymes involved in this pathway have become attractive candidates for anti-cancer drug design.

The phosphoribosylaminoimidazole carboxylase, phosphoribosylaminoimidazole succinocarboxamide synthetase (*PAICS*) gene encodes a bifunctional enzyme that has phosphoribosylaminoimidazole carboxylase activity in its N-terminal region and phosphoribosylaminoimidazole succinocarboxamide synthetase activity in its C-terminal region [3]. This enzyme catalyzes steps 6 and 7 of the de novo purine biosynthesis pathway (Phosphoribosyl pyrophosphate amidotransferase (PPAT) catalyses step 1; phosphoribosylglycinamide formyltransferase (GART) steps 2, 3 and 5; phosphoribosylformylglycinamidine synthase (PFAS) step 4; phosphoribosylaminoimidazole carboxylase (PAICS) steps 6 and 7; adenylosuccinate lyase (ADSL) step 8 and 5-aminoimidazole-4-carboxamide ribonucleotide formyltransferase/IMP cyclohydrolase (ATIC) steps 9 and 10) [4]. Thus, PAICS is a promising target for rational anticancer drug design [3]. Alterations in PAICS expression in humans are associated with various types of cancer. Through a functional yeast survival screen of tumor-derived cDNA libraries, PAICS was shown to be overexpressed in certain tumor types and capable of suppressing apoptosis in human cells [5]. Immunohistochemical analysis confirmed the overexpression of PAICS in human melanoma samples compared to expression in normal skin biopsies [5]. High PAICS expression was also found in human lung squamous cell carcinoma by using suppression subtractive hybridization [6]. PAICS also showed a general association with progression-free glioblastoma survival [7]. Moreover, mutations in PAICS have been reported in human melanoma through whole-exome sequencing and SNP array profiling of six cutaneous melanoma cell lines derived from metastatic patients [8]. Furthermore, analysis of the *Oncomine* database demonstrated that the *PAICS* gene is significantly overexpressed in a variety of tumors, including colorectal cancer, brain and CNS cancer, bladder cancer, and lymphoma. To date, PAICS has been found to affect breast cancer cells growth (), however, how it affects cell cycle has not been studied [9].

Using lentivirus-based RNA interference (RNAi) we knocked down the endogenous expression of PAICS in the human breast cancer cell lines ZR-75-30 and MDA-MB-231 to explore whether it is involved in breast cancer growth. In addition, the effects of PAICS silencing on cell viability, proliferation, cell cycle progression, and apoptosis were further investigated.

## Methods

### Materials

RPMI-1640 (#SH30809.01B+) and DMEM (#SH30243.01B+) were obtained from Hyclone (Logan, Utah, USA). Fetal bovine serum (FBS; #04-001-1A) was obtained from Biological Industries (BI; Israel). Lipofectamine 2000 and TRIzol^®^ Reagent were obtained from Invitrogen (Carlsbad, CA, USA). M-MLV Reverse Transcriptase was obtained from Promega (Madison, WI, USA). All other chemicals were from Sigma-Aldrich (St. Louis, MO, USA). The lentiviral vector (pGP-L) and packaging vectors (pVSVG-I and pCMVΔR8.92) were purchased from Shanghai Hollybio (Shanghai, China). Rabbit anti-human PAICS (polyclonal, 1:1000, #12967-1-AP), rabbit anti-human GAPDH ((polyclonal, 1:500,000, #10494-1-AP), rabbit anti-human CDK4 (polyclonal, 1:500, #11026-2-AP), mouse anti-human Cyclin D1 (monoclonal, 1:1000, #60178-1-1g), mouse anti-human Bcl2 (monoclonal, 1:500, #60178-1-1g), rabbit anti-human Bcl-xl (polyclonal, 1:500, #10783-1-AP), rabbit anti-human cleaved caspase 3 (polyclonal, 1:500, #25546-1-AP), and rabbit anti-human Cyclin E (polyclonal, 1:500, #11554-1-AP) antibodies were obtained from Proteintech Group, Inc. (Chicago, IL, USA); horseradish peroxidase-conjugated goat anti-rabbit (polyclonal, 1:5000, #SC-2054) secondary antibody was from Santa Cruz (Dallas, TX, USA). Rabbit anti-human P21 (monoclonal, 1:1000, #2947) and rabbit anti-human PARP (polyclonal, 1:1000, #9542) antibodies were purchased from Cell signaling (Boston, MA, USA). The Annexin V-APC/7-AAD Apoptosis Assays Kit (#KGA1026) was purchased from Mybioreagent.Ltd (KeyGEN BioTECH, China).

### Cell culture

ZR-75-30 and MDA-MB-231 human breast cancer cell lines and 293T human embryonic kidney cells were purchased from the Cell Bank of Chinese Academy of Science (Shanghai, China). ZR-75-30 and MDA-MB-231 cells were maintained in RPMI-1640, and 293T cell lines were maintained in DMEM, both supplemented with 10% FBS, at 37 °C in a humidified atmosphere of 5% CO_2_.

### Lentivirus-delivery of silence of PAICS

The siRNA sequence targeting human *PAICS* (NCBI accession number: NM_001079525) was 5′-GCTGCTCAGATATTTGGGTTA-3′, which was subjected to BLAST analysis against the human genome database to mitigate off-target silencing of other genes. A fragment (5′-TTCTCCGAACGTGTCACGT-3′) with no significant homology to mouse or human gene sequences was used as a negative control. siRNA sequences were synthesised as shRNA form (siRNA sense-loop-antisense). shRNAs were cloned into the pGP vector, which was then transfected into 293T cells with packaging vectors (pVSVG-I and pCMVΔR8.92) using Lipofectamine 2000 according to the manufacturer’s instructions. The supernatant was collected 48 h later, centrifuged (4000×*g*, 4 °C, 10 min) to remove cell debris, filtered through 0.45-μm cellulose acetate filters, and then concentrated again (4000×*g*, 4 °C, 15 min). ZR-75-30 cells were dispensed into 6-well plates at a density of 30,000 cells per well and transduced with shRNA-expressing lentivirus (siCon or siPAICS) at a multiplicity of infection (MOI) of 35. MDA-MB-231 cells were dispensed into 6-well plates at a density of 40,000 cells per well and transduced with shRNA-expressing lentivirus (siCon or siPAICS) (MOI 30). The lentiviral vectors expressed green fluorescence protein (GFP), which allowed for the measurement of infection efficiency in transduced cells.

### Quantitative real-time PCR analysis

ZR-75-30 and MDA-MB-231 cells were harvested 5 days after lentivirus transduction. Total cellular RNA was extracted using Trizol reagent and reverse transcribed to cDNA using M-MLV reverse transcriptase, according to the manufacturer’s instructions. qPCR products were detected with SYBR Green using the BioRad Connet Real-Time PCR platform (Shanghai, China). The qPCR procedure included denaturation at 95 °C for 1 min, 40 cycles of denaturation at 95 °C for 5 s and extension at 60 °C for 20 s. *β*-*actin* was amplified as an internal control. Relative quantitation was analyzed by calculating the difference ΔC(T) between the C(T) of *β*-*actin* and the C(T) of the target gene and by computing the 2^−ΔΔC(T)^ value. The primers used in this study were summarized as Table 1.

**Table 1:** The primers used in this study

GENE	Forward	Reverse
*PAICS*	5′-CTGAAGGGCTCCAAATGGTA-3′	5′-GGTCCTTTATGCGCAGATGT-3′
*β*-*actin*	5′-GTGGACATCCGCAAAGAC-3′	5′-AAAGGGTGTAACGCAACTA-3′

### Western blotting

ZR-75-30 and MDA-MB-231 cells were harvested 5 days after lentivirus transduction. Total protein was extracted with 2× SDS sample buffer (100 mM Tris–HCl (pH 6.8), 10 mM EDTA, 4% SDS, 10% Glycine). Equal amounts of lysate (30 μg) per lane, as determined by bicinchoninic acid (BCA) assays, were separated by 10% SDS-PAGE and transferred to PVDF membranes. The membranes were blocked with 5% nonfat dry milk in tris-buffered saline with tween 20 (TBST) for 1 h at room temperature, and then incubated with primary antibody overnight at 4 °C. The membranes were washed three times with TBST and then incubated with secondary antibody for 1 h at room temperature. The blots were detected using an enhanced chemiluminescence (ECL) kit (Amersham, Beijing, China) and visualized by exposure to X-ray film. GAPDH was used as a control to verify equal protein loading.

### MTT viability assay

To evaluate the effect of PAICS on breast cancer cell viability, a 3-(4,5-dimethylthiazol-2-yl)-2,5-diphenyl-tetrazolium bromide (MTT) colorimetric assay was performed using ZR-75-30 and MDA-MB-231 cells 3 days after lentivirus transduction. Briefly, ZR-75-30 and MDA-MB-231 cells were separately dispensed into 96-well plates at a concentration of 2000 per well. The plates were incubated for 1–5 days at 37 °C. On each day, 20 μl of MTT solution (5 mg/ml) was added and incubated for 4 h. Afterwards, acidic isopropanol (10% SDS, 5% isopropanol and 0.01 M HCl) was added at a volume of 100 μl per well and incubated overnight at 37 °C. The absorbance at 595 nm of each well was determined using an Microplate Reader (Epoch, VT, USA).

### Colony formation assay

To evaluate the effect of PAICS on long-term proliferation of breast cancer cells, colony formation assays were performed on ZR-75-30 cells 4 days after lentivirus transduction. Briefly, ZR-75-30 cells were dispensed into 6-well plates at a concentration of 500 per well. The culture medium was changed every 3 days. ZR-75-30 cells were cultured for 6 days until the biggest single colony contained greater than 50 cells. The colonies were stained with crystal violet for 20 min, washed with water, and then air-dried. Cell colonies were captured and counted using a microscope (Olympus, Japan).

### Fluorescence-activated cell sorting (FACS) analysis

To evaluate the effect of PAICS on breast cancer cell cycle progression, flow cytometry was performed on ZR-75-30 cells 7 days after lentivirus transduction. Briefly, ZR-75-30 cells were dispensed into 6-cm dishes at a concentration of 40,000 per dish. After culturing at 37 °C for 6 days, cells were harvested, fixed in 70% ethanol, and stored overnight at 4 °C. The cells were then treated with NaCl/Pi staining solution (50 µg/ml PI (propidium iodide) and 100 µg/ml RNase A). Following incubation for 1 h in the dark at room temperature, cells were analyzed by flow cytometry (FACSCalibur; Becton–Dickinson, San Jose, CA, USA). Fractions of cells in G0/G1, S, and G2/M phases were analyzed with dedicated software (ModFit LT, ME, USA).

### Flow cytometric analysis of apoptosis

The quantification of apoptotic cells was determined by flow cytometry using an AnnexinV/7-AAD double staining Kit according to the manufacturer’s instructions. Briefly, ZR-75-30 cells infected with siPAICS or siCon were seeded separately in 6 cm dishes at 1 × 10^5^ cells/dish. When the cells reached approximately 80% confluence, they were harvested, washed twice with PBS, and suspended in 450 μl binding buffer; Annexin V was then added at room temperature. The cells were stained for 15 min in the dark and were then resuspended in 450 μl binding buffer. Cells were also stained with 7-AAD in the dark. Cell apoptosis was analyzed using a Gallios flow cytometer (Beckman, CA, USA).

### Statistical analysis

Statistical analysis was performed using GraphPad Prism 5.0. All data were expressed as mean ± standard deviation (SD). Differences between two groups were analyzed by a Student’s *t* test, and a p value less than 0.05 was considered statistically significant.

## Results

### Knockdown of PAICS expression with lentivirus-delivered shRNA

**Fig. 1:**
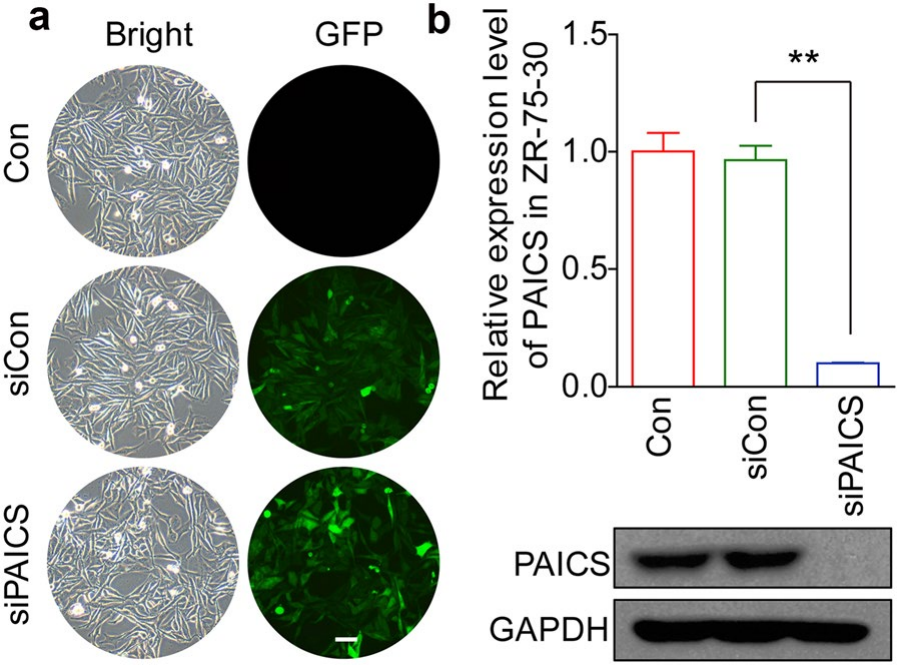
Lentivirus-delivered shRNA targeting PAICS depletes endogenous expression in ZR-75-30 breast cancer cells. **a** Evaluation of the lentivirus transduction rate, which was more than 80% as estimated by fluorescence and light microscopy observations. **b** Quantitative analysis of PAICS knockdown efficiency in ZR-75-30 cells assessed by qRT-PCR. The *β*-*actin* gene was used as an internal control. And representative immunoblot showing PAICS knockdown efficiency determined. GAPDH was used as an internal control. Data are shown as mean ± SD (n = 3; t test). **p < 0.01; magnification, ×100

ZR-75-30 and MDA-MB-231 cells were transduced with shRNA-expressing lentivirus (siCon or siPAICS). GFP expression was observed by fluorescent microscopy 3 days post-transduction. As depicted in Figs. 1a and 3a, more than 80% of cells expressed GFP, as indicated by green fluorescence in siCon and siPAICS groups, indicating successful infection. The inhibitory effect of PAICS shRNA on its endogenous expression in ZR-75-30 and MDA-MB-231 cells was separately examined by qRT-PCR and western blotting. As depicted in Figs. 1b and 3b, the mRNA level of *PAICS* was significantly reduced in both cell lines with siPAICS infection compared to expression in siCon-transduced cells. Immunoblotting further verified the ablation of PAICS expression at the protein level. Therefore, lentivirus-delivered shRNA could specifically deplete endogenous PAICS expression in ZR-75-30 and MDA-MB-231 cells.

### Effect of PAICS knockdown on cell proliferation and colony formation

**Fig. 2:**
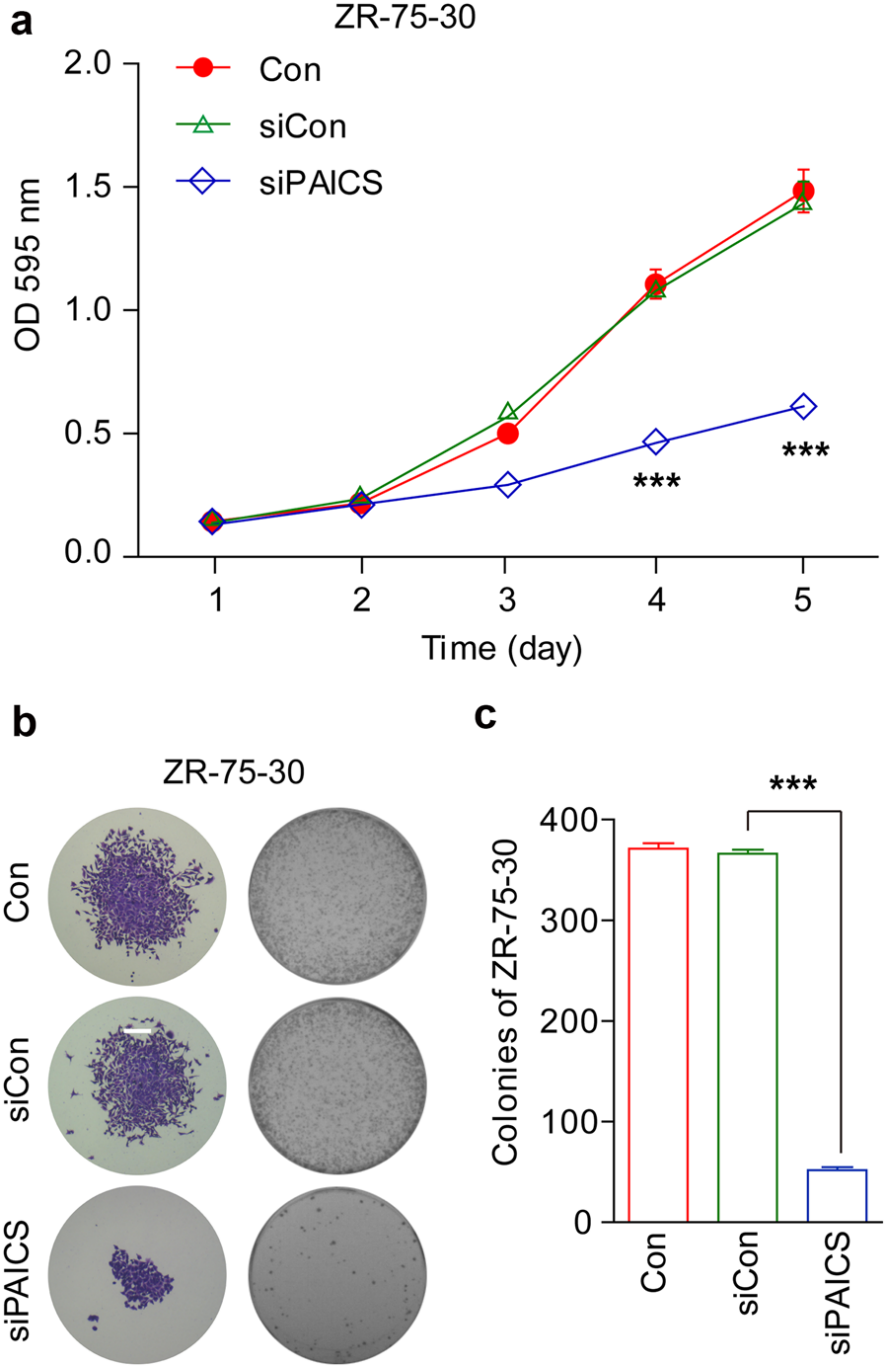
Knockdown of PAICS inhibits viability and proliferation of ZR-75-30breast cancer cells. **a** MTT assays showing growth curves of ZR-75-30cells. The number of viable cells was substantially decreased in the siPAICS group compared to that in the siControl (siCon) and control (Con) group. **b** Representative colony formation showing clonogenic survival determined in ZR-75-30 cells. **c** The number of colonies was substantially decreased in the siPAICS group compared to that in the siCon group and the Con group. Data are shown as mean ± SD (n = 3; t test). ***p < 0.001; magnification, ×40

**Fig. 3:**
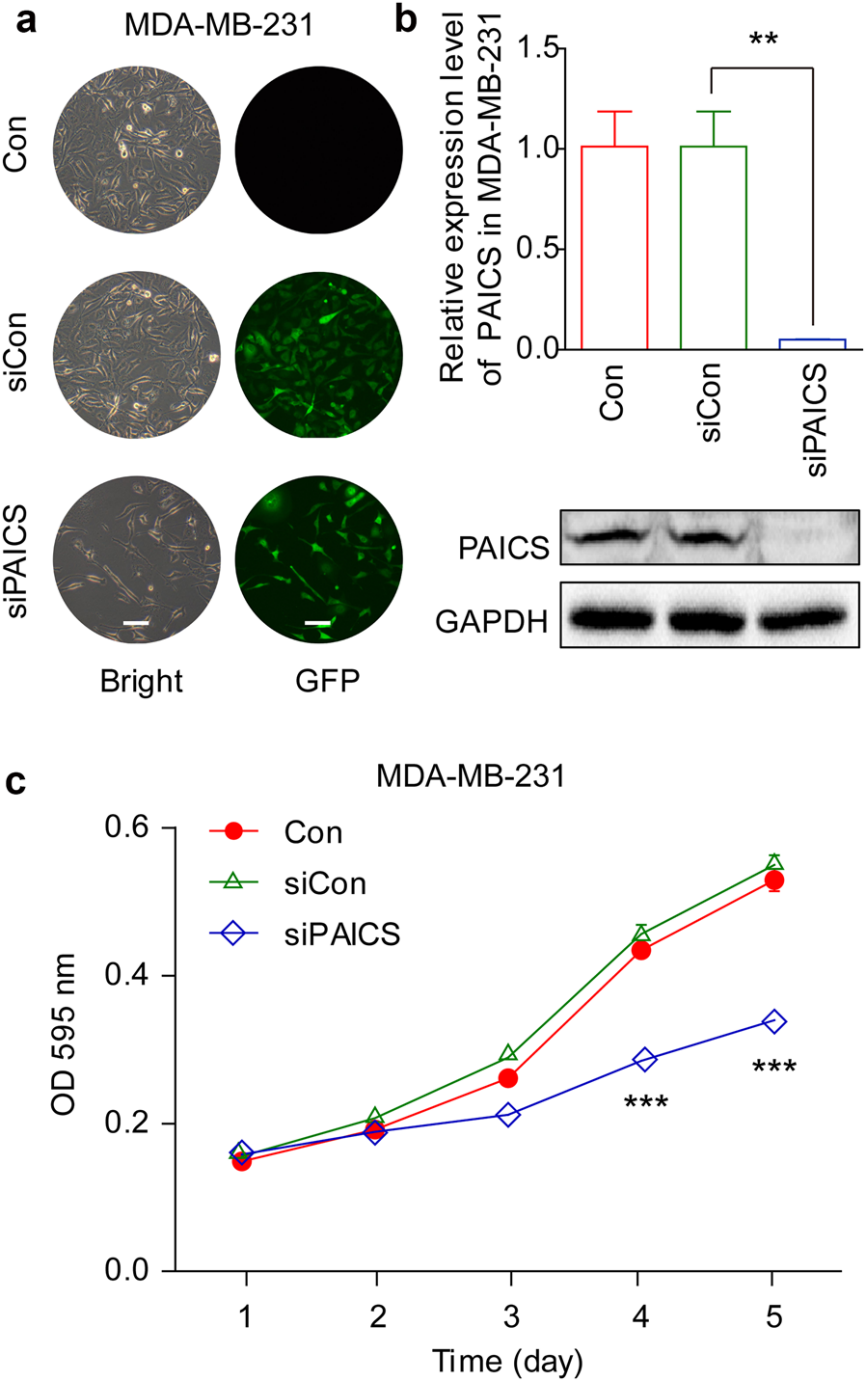
Effect of PAICS endogenous depletion on MDA-MB-231. **a** The lentivirus transduction rate (more than 80%) as observed under fluorescence and light microscopy. **b** Quantitative qPCR and western blot showed the efficient knonkdown of PACIS in mRNA and protein level in ZR-75-30 cells. **c** MTT assays ompared growth curves of MDA-MB-231, before and after PAICS depletion. **p < 0.01; magnification, ×100

We next examined the effects of PAICS knockdown on the viability and proliferation of ZR-75-30 and MDA-MB-231 cells. After infection with PAICS shRNA, MTT assays were performed on both cell types for 5 consecutive days. As depicted in Figs. 2a and 3c, the siPAICS reduced the number of viable cells as compared to that with siCon and Con (cells that were not transduced) conditions; specifically, a reduction of 57.5% (ZR-75-30) and 38.2% (MDA-MB-231) was observed on day 5 (p < 0.001). In addition, colony formation assays were performed to evaluate the impact of PAICS silencing on cell proliferation in ZR-75-30 cells. Crystal violet staining showed that the maximum size of each colony was smaller and the total number of colonies was fewer in siPAICS-infected cells compared to those in siCon or Con conditions (Fig. 2b). Thus, colony formation ability was significantly impaired, and was specifically reduced by 85.8% in ZR-75-30 cells after PAICS knockdown (p < 0.001, Fig. 2c). These data indicate that knockdown of PAICS strongly reduces viability and proliferation of breast cancer cells.

### Effect of PAICS knockdown on cell cycle progression

**Fig. 4:**
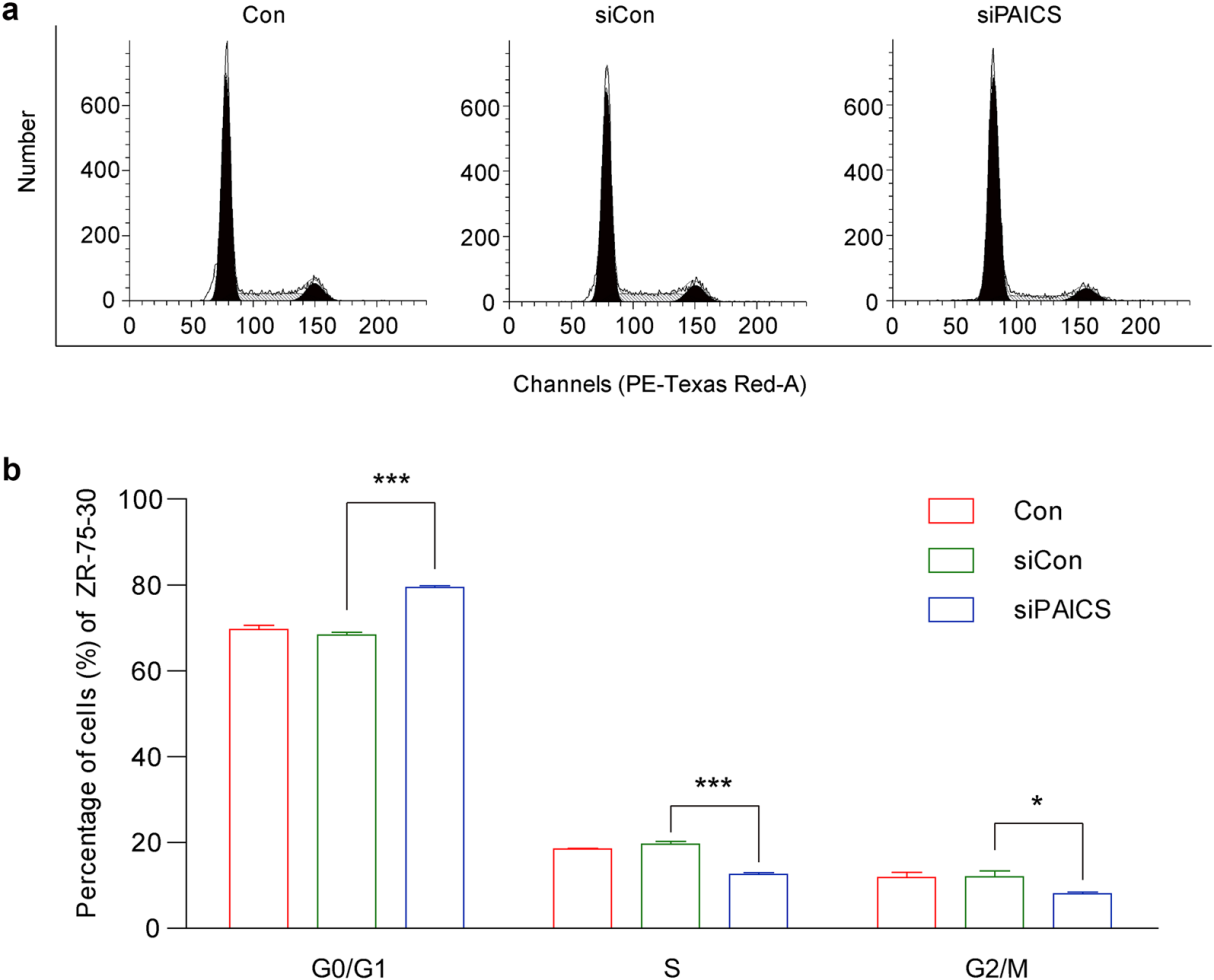
Knockdown of PAICS arrests cell cycle progression in ZR-75-30 breast cancer cells. **a** Comparison of the cell population in G0/G1, S, and G2/M phases between siControl (siCon) and siPAICS groups, as assessed by flow cytometry. **b** The percentage of cells in G0/G1 phase was significantly higher in the siPAICS group than in the siCon group, whereas the proportion of cells in the S phase was simultaneously reduced. Data are shown as mean ± SD (n = 3; t test). ***p < 0.001

To test the mechanism through which PAICS modulates cell proliferation, flow cytometry assays were performed to assess the cell cycle progression of ZR-75-30 cells. Representative images of cell cycle distribution are presented in Fig. 4a. As depicted in Fig. 4b, the percentage of cells in the G0/G1 phase increased from 68.34 ± 0.70% in siCon-infected cells to 79.47 ± 0.45% in siPAICS-infected cells. In contrast, the percentage of cells in S phase decreased from 19.61 ± 0.66% in siCon-infected cells to 12.53 ± 0.40% in siPAICS-infected cells. This indicated that PAICS shRNA strongly blocks (p < 0.001) cell cycle progression of ZR-75-30 cells. Taken together, these results suggest that knockdown of PAICS inhibits ZR-75-30 cell growth by inducing G0/G1 phase cell cycle arrest.

### Knockdown of PAICS promotes apoptosis in breast cancer cells

**Fig. 5:**
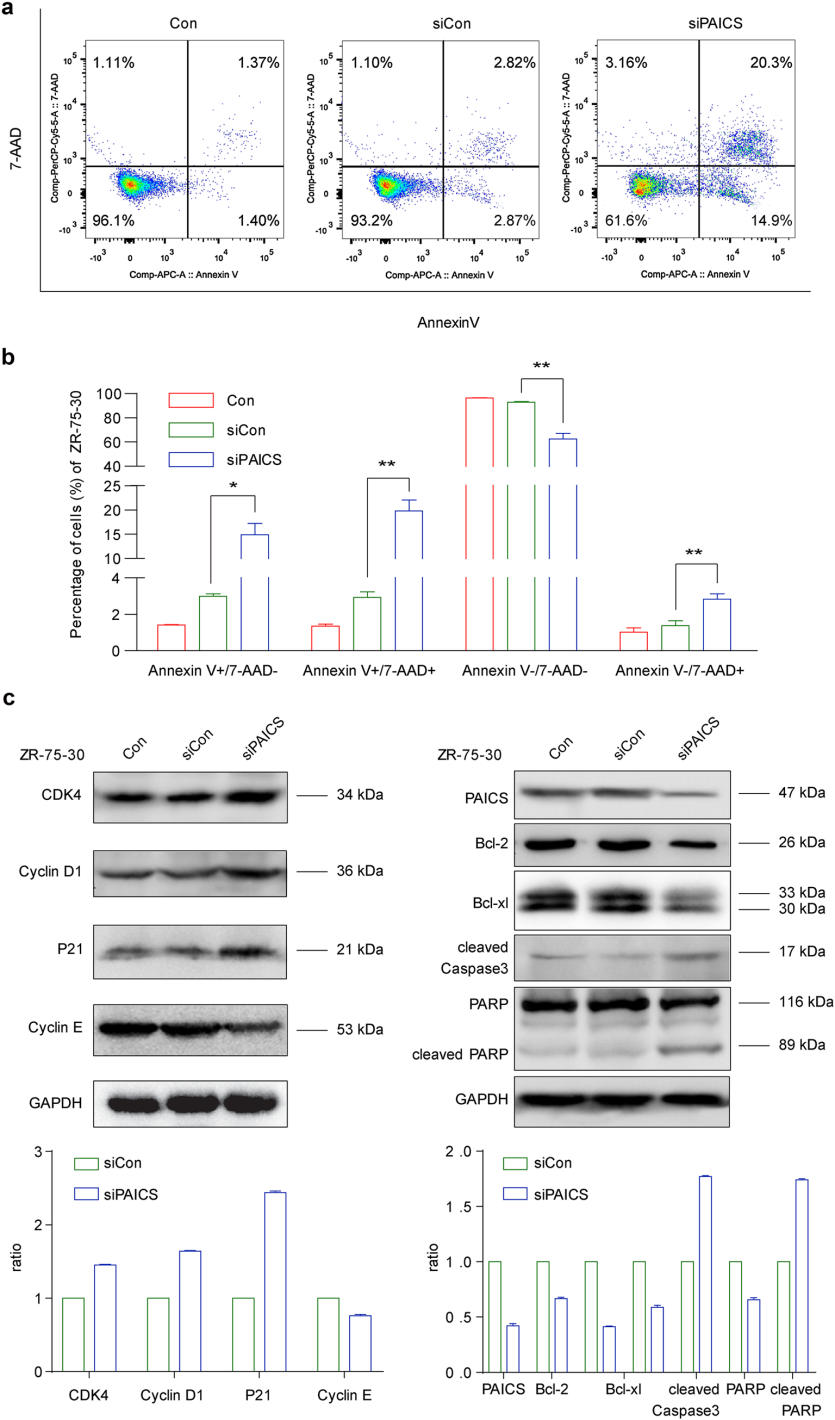
Assessment of apoptosis after PAICS knockdown in ZR-75-30 breast cancer cells. Apoptosis was investigated by flow cytometric analysis. **a** Representative diagrams showing the analysis of apoptosis. **b** The percentages of AnnexinV-APC/7-AAD-positive apoptotic cells are presented. **c** Cell cycle-related and apoptosis-related molecules were detected by western blotting using specific antibodies after PAICS knockdown in ZR-75-30 cells. GAPDH was used as the loading control. Data are shown as mean ± SD (n = 3; t test). ***p < 0.001

Flow cytometry using Annexin V- and 7-AAD-stained ZR-75-30 cells was performed to quantitatively determine the percentage of apoptotic cells after different treat-ments. As shown in Fig. 5a, b, PAICS knockdown increased apoptosis in ZR-75-30 cells. The early (14.9%) and late (20.3%) apoptosis rates of siPAICS-infected ZR-75-30 cells was increased compared to those in siCon-infected ZR-75-30 (2.87 and 2.82%) (early apoptosis, p < 0.05; late apoptosis, p < 0.01) and Con (1.43 and 1.37%) cells.

### Knockdown of PAICS regulates cell cycle and apoptotic markers

To speculate on mechanisms through which PAICS knockdown promotes cell cycle arrest and increases apoptosis, we examined alterations in the expression of cell cycle regulators and markers of apoptosis. As shown in Fig. 5c, the expression levels of CDK4 and CyclinD1, associated with G1-S transition, were increased in the siPAICS group. In addition, the expression levels of cleaved-caspase 3 and cleaved-PARP were increased, whereas the anti-apoptotic proteins Bcl-2 and Bcl-xl were downregulated in shPAICs groups (Fig. 5c).

## Discussion

Breast cancer is the most frequently diagnosed cancer and the leading cause of cancer-related death among females in less developed countries [10]. The incidence of breast cancer is increasing at a surprisingly rapid pace [11], 1.67 million new breast cases were diagnosed and 52.9% of which was in developing countries in 2012, compared to only 35% in 1980 [12]. According to American Cancer Society estimates, breast cancer alone is expected to account for 29% of all new cancers in women in 2015, which far exceeds the proportion of the second most common malignancy, lung and bronchus cancer (13%) [13]. However, the death rate in females with breast cancer is down by 35% from peak rates as a result of improvements in early detection and treatments [14]. Although early detection, precise resection using wide margins, and systematic adjuvant therapy have improved survival, their efficacy is beginning to plateau [15]. A series of molecular biology studies have identified that breast cancer is a genetic disease. It has become clear that the activation of oncogenes and the inactivation of tumor-suppressive genes are involved in the regulation of the cellular physiological processes that lead not only to abnormal cell proliferation and differentiation, but also to defective apoptosis and drug resistance [16, 17]. Biomarkers are attracting increasing attention as potential predictors of breast cancer patient survival [18, 19]. Although alterations in many oncogenic and tumor-suppressive genes are reportedly implicated in breast cancer, the molecular mechanisms through which the proliferation of malignant tumor cells is maintained remains poorly understood. Therefore, it is of great importance to identify novel therapeutic targets for the prevention and treatment of breast cancer.

In the present study, we primarily identified that the *PAICS* gene, which encodes an enzyme required for de novo purine biosynthesis, might be a potential tumor marker in human breast cancer. Knockdown of endogenous PAICS expression by shRNA-expressing lentivirus significantly decreased the viability and proliferation of ZR-75-30 breast cancer cells. Moreover, FACS analysis showed that knockdown of PAICS induced significant arrest in the G0/G1 phase of the cell cycle, which was important for the inhibition of cell proliferation. CDK4 and Cyclin D1 are key regulators for G1-S transition during the cell cycle. Cyclin D1 is an M-phase-promoting factor and is essential for the initiation of mitosis [20]. We found that the expression levels of CDK4 and CyclinD1 were increased in PAICS-silenced cells. It is reasonable to speculate that the mechanisms of PAICS knockdown-mediated inhibition of G1-S transition might be partly be through the suppression of CDK4 and CyclinD1. Therefore, it is likely that PAICS can modulate breast cancer growth via the regulation of a subset of cancer-related genes involved in G1-S checkpoint progression. Flow cytometry analysis also revealed that PAICS knockdown by shRNA induced apoptosis. The presence of cleaved PARP is one of the most commonly used diagnostic tools for the detection of apoptosis in many cell types [21]. Significant proteolytic cleavage of PARP was detected in ZR-75-30 breast cancer cells infected with siPAICS by western blotting, indicating an anti-apoptotic role for PAICS. Apoptosis is a type of programmed cell death that is caspase dependent [22]. Moreover, the expression of cleaved-caspase 3 was obviously increased in PAICS-silenced cells. In addition, the anti-apoptotic mitochondrial proteins Bcl-2 and Bcl-xl were decreased in ZR-75-30 breast cancer cells infected with siPAICS. Therefore, we can conclude that the growth inhibitory effect of PAICS silencing in breast cancer cells was probably due to the induction of mitochondrial-related apoptosis. Whether inhibition of de novo purine biosynthesis is associated with growth suppression in ZR-75-30 breast cancer cells after silencing of PAICS has not been proven in this study. Further investigation is required to reveal the molecular exact basis for the oncogenic function of PAICS in breast cancer cells.

## Conclusions

In summary, knockdown of PAICS expression by RNAi significantly inhibits the growth of ZR-75-30 cells by inducing cell cycle arrest. Our findings demonstrate for the first time that PAICS plays an essential role in breast cancer cell growth, which might be useful for the identification of novel targets for human breast cancer therapy.

## Abbreviations

PAICS: phosphoribosylaminoimidazole carboxylase, phosphoribosylaminoimidazole succinocarboxamide synthetase
shRNA: short hairpin RNA
RNAi: RNA interference
GFP: green fluorescence protein
BCA: bicinchoninic acid
TBST: tris-buffered saline with tween 20
ECL: enhanced chemiluminescence
MTT: 3-(4,5-dimethylthiazol-2-yl)-2,5-diphenyl-tetrazolium bromide
FACS: fluorescence-activated cell sorting
SD: standard deviation
